# Supplementary material for: Rotavirus and bacterial diarrhoea among children in Ile-Ife, Nigeria: Burden, risk factors and seasonality
Source: PLoS One. 2023 Sep 12;18(9):e0291123. doi: 10.1371/journal.pone.0291123 (PMC10497142; doi:10.1371/journal.pone.0291123)
Supplement: S1 File — (DOCX) [file pone.0291123.s001.docx]

**Supporting Information**

The authors wish to state that the minimal data set required to replicate all study findings are reported in the article, as well as related methods. All data upon which mean, standard deviations and conclusion were based are available in the manuscript.
