# Supplementary material for: Rotavirus and bacterial diarrhoea among children in Ile-Ife, Nigeria: Burden, risk factors and seasonality
Source: PLoS One. 2023 Sep 12;18(9):e0291123. doi: 10.1371/journal.pone.0291123 (PMC10497142; doi:10.1371/journal.pone.0291123)
Supplement: Attachment — Submitted filename: reviewer letter_01OCT2022.docx [file pone.0291123.s002.docx]

Dear Academic Editor, PLOS ONE

Thank you for your invitation to review the manuscript “Rotavirus and bacterial diarrhoea among children in Ile-Ife, Nigeria: burden, clinical/environmental factors and seasonality”. The manuscript describes a basic hospital investigational of diarrhoea. There are several issues in the manuscript that require further elaboration or at least clarification:

1. **Title**

*Consider to review the manuscript title, because it does not reflect what has been done as methodology and main findings, to be more concise and suggestive.*

2. **Abstract**

The abstract is too long, needs to be shorten and synthetized. Additionally, seems to be missing the justification for conducting the study. There are many statements, which needs to be reviewed:

“Pathogens were detected using ELISA, conventional cultural technique and PCR. *Escherichia coli* (*E. coli*) isolates were pathotyped”: *Was the pathotyping method different from PCR? Please, specify which pathogen was detected by which technique.*

“etiology”: *Please standardize, sometimes it appears aetiology.*

“E. coli. was the most common bacteria etiology with high multidrug resistance in the DEC isolates”: *Needs to be corrected. Considering that E. coli can also be commensal, the authors should only consider the frequency of DEC and classification by each pathotype rather the overall frequency of E. coli isolation.*

“Variation in seasonal peaks of viral and bacterial etiology of diarrheoa”: *This should be interpreted with caution because the figure of seasonality (Fig. 3) only describes rotavirus and E.coli.*

“The E. coli pathotypes detected were Shiga Toxin E. coli (STEC), enterohaemorrhagic E. coli (EHEC) and enteroaggregative E. coli (EAEC)”. *Why the author considered STEC and EHEC as distinct pathotypes. What is the difference between both? Your consideration for the papers below regarding the classification for this pathotype.*

**Delannoy, S., Beutin, L., and Fach, P. (2013). Discrimination of Enterohemorrhagic Escherichia coli (EHEC) from Non-EHEC Strains Based on Detection of Various Combinations of Type III Effector Genes. *J. Clin. Microbiol.* 51, 3257–3262. doi: 10.1128/JCM.01471-13.**

**Orth, D., and Wurzner, R. (2006). What Makes an Enterohemorrhagic Escherichia coli? *Clin. Infect. Dis.* 43, 1168–1169. doi: 10.1086/508207.**

3. **Introduction**

*The global burden of diarrhoea should be updated with recent data, the authors present data from 5 years ago ((WHO 2017; UNICEF 2018).*

Statements:

“Considering the immaturity of children’s immune system, these poor living conditions increase the vulnerability of children younger than 5 years to diarrhoea when exposed to pathogens that cause diarrhoea”: *Please review the sentence.* “(rotaviruses, caliciviruses, noroviruses, and sapoviruses)”: *Noroviruses and sapoviruses are Caliciviruses.*

“or having contact pathogen carriers”: *Please clarify what does this statement means.*

“Western states due to the level of enlightenment”: *What does enlightenment means and relation to pathogens?*

“Nigeria being a tropical country has two main seasons: the dry season which lasts from October until around April (or March in some parts of the country), determined by high temperatures and low humidity and the wet or rainy season which is for the remaining months of the year”: *What is the relevance to bring Nigeria season in the introduction? It should be moved to methodology.*

The authors should consider mentioning in the justification that there is lack of data regarding diarrhoea aetiology in Nigeria and that the study is bringing some data to fill this gap

“*Escherichia coli* (ETEC), enteropathogenic *Escherichia coli* (EPEC), *Shigella species, Samonella species, Campylobacter jejuni*), and protozoa or parasites (*Entamoeba histolytica, Giardia lambia*)”. *Correct the taxonomy for Salmonella spp.*

4. **Materials and Methods**

*In general authors should review profoundly the study design, there is some key information missing, like description of inclusion criteria, definition of acute gastroenteritis, sample collection and storage.*

Specifically, in:

2.1. Study design: “Informed consent was taken from the caregivers of all the study participants, and children who have taken antibiotics within 10 days of presentation were excluded from the study. *Why did the authors considered excluding children who took antibiotics in just 10 days and not more? How did the authors ensure that the caregiver recognized whether the child received an antibiotic or other unrelated medication?*

2.2 *Ethics*”

Ethics Approval: “Written informed consent was taken from caregivers. A semi-structured interviewer administered questionnaire was administered to caregivers of study participants, detailing relevant sociodemographic information, clinical and environmental factors”: *This should not be part of the ethical section.*

*Why did the authors not perform AST for Salmonella and Shigella?*

DNA Extraction: “The DNA of the bacteria”: *Should not be E.coli colonies? As PCR was only performed for its pathotypes.*

“The plates were incubated for 24 hrs. aerobically at 37 °C. Distinct colonies were picked

and identified by standard biochemical tests.” *What was the procedure for those cultures with no growth within 24 hrs, the plates were reincubated?*

“The isolated organisms suspected to be *E. coli* by their cultural and biochemical characteristics

were confirmed as *E. coli* by polymerase chain reaction (PCR) using primers specific to *E. coli*

16S rRNA gene, adopting the procedure described by Mamun *et al*. (2016).” *This reference is not the appropriate. Please consider to add appropriate reference. In addition, specify a little bit more about the primers and protocols used.*

“All the *E. coli* isolates were screened for virulence genes of five different pathotypes of diarrhoeagenic *E. coli,* including enterotoxigenic *E. coli* (ETEC) and enteroinvasive *E. coli* (EIEC), enteropathogenic *E. coli* (EPEC), enteroaggregative *E. coli* (EAEC), and enterohaemorrhagic *E. coli* (EHEC) as previously described by Aranda *et al*. (2004) with slight modifications*.*” *Mention the targets used for DEC detection. Which modifications were made? Describe more.*

“The disc diffusion method was used for the antibiotic susceptibility testing of DEC isolates on

Mueller Hinton agar. Commonly used and available antibiotics (Oxoid Ltd, UK) impregnated…….” *Which antibiotics were used for AST. Are they common used in Nigeria? Why the authors only tested for 6 antibiotics?*

“After incubation, the plate was well aspirated and washed 5 times using the wash….”*The washing was manual or automatized?*

“Finally, 50 μl of stop solution was added to all the wells and the result read on ELISA plate reader at 450”. *Provide the equipment description.*

Rotavirus Screening: *Please if ELISA steps are described in the manufacture'r protocol, please resume.*

Statistical Analysis: *Please review the meaning of the statistical package (SPSS).*

“Student T-test and Chi-square”: *P value is used to compare and test the significance of your hypothesis and not to draw conclusions but to suggest what may be the factors.*

5. **Results**

*In general, the authors should consider bringing a table describing the general characteristics of the population before presenting specific analysis, also adding information related to the virulence factors. Additionally, all the subtitles should be reviewed, authors describe them as if they were giving a conclusion or the main finding, ignoring the whole data in the section. All the figure quality should be improved with programs with better resolution. In all the table and images should be added the place, kind of population and study period. There are many results without specifying the correspondent table/figure, please review. There are also so many redundancies.*

“Rotavirus more prevalent than bacterial in mono or multiple infection”: *Please review the numbers and percentages, sometimes it appears n (%) without a denominator while sometimes both*. *Review the terms: single co-infections, double, triple, four, which the pathogens are not specified in the section.*

“Majority of the pathotyped isolates belong to the Shiga Toxin E. coli (STEC)”: *Please specify or explain more what does cross bands means.*

Multidrug resistance observed in the DEC isolates: *Please indicate how many isolates were tested ans? Also present the resistance according to the pathotypes.*

Clinical and environmental factors associated with rotavirus diarrhoea: “samples with quantities not enough for both bacterial and viral screening were excluded”: *This should have been mentioned in the methods section.*

Table 2: The clinical and risk factors should be presented separately. *Specify if the numbers in brackets are numbers or percentages.*

Rotavirus infection occurred only in the dry season: “Samples were not collected in December 2019 and March 2020 due to the outbreak of the Lassa fever virus in Nigeria and the outbreak of the global coronavirus pandemic which brought about restrictions in gaining access to the hospital and made parents to avoid coming to the hospital”: *This information should have been described in the method section and authors should adjust the study period. This information would also be cited as study limitation.*

“At least one pathogen was detected from 63 (60.6%) of the children having gastroenteritis from

the one hundred and four children enrolled in this study.” *In the abstract the authors mentioned that only 90 children had sufficient samples for rotavirus screening. Now the author considered all the 104 children. Please check.*

*The AST data should be complemented with detection of resistant determinants.*

“In multiple pathogen infection, the prevalence of RVA, *E.coli*, *Shigella spp.,* and *Salmonella spp.* was 45% (41/91), 23.1% (24/104), 21.2% (22/104) and 10.6% (11/104) respectively……….”

*When the authors mentioned E. coli are referring to all DECs or all E. coli isolated by conventional microbiology? The authors should only consider DECs for analysis.*

“….genes. Sixteen (69.6%) of the isolates were STEC, 2 (8%) were EHEC, one isolate (4%) was……” *What is the difference between STEC and EHEC?*

*“*Multidrug resistance observed in the DEC isolates*”. All this section should be rewritten, there are so many redundancies.*

“Multidrug resistance (the resistance of an organism to 3 or more different classes of antibiotics)”. *This sentence should be moved to methodology.*

*“*Table 1: distribution of Single and Multiple infections according to age.*”Consider to include %s.*

6. **Discussion**

*Overall, the discussion is too long, the authors should summarize the text and focus on their results first, before referencing similar findings.*

Minor comments:

*Please give more recent publication for comparisons of diarrhoea aetiology in Nigeria, (Ogunsanya et al., 1994). The authors should even use publication referring single diarrhoea pathogens detection.*

“Niger, Burkina Faso, Nicaragua, India, and Turkey where viral and bacterial gastroenteritis prevalence of 70%, 64%, 61.1%, 60%, and 59.2% were reported respectively” *Did the referenced studies use the same detection method? Here the authors do not specify the proportion of viral and bacterial detection singularly.*

“Rotavirus infection was observed in this study to have the highest prevalence among the different microbial etiology of childhood diarrhoea screened for”: *This sentence seems incomplete*.

“This might not be unconnected with the fact that majority of children are not vaccinated against rotavirus infection and the environmental factors in which they live aids the spread of the virus”: *As Nigeria vaccine was launched in August 2022, the verb mode should be reviewed. How do the authors conclude that the environmental factors would spread the virus? Which tests were performed to test this conclusion?*

“In this study, a slight reduction in the prevalence of rotavirus gastroenteritis was observed (45.1%) compared to the report by Japhet et al. (2018) (47.6%) showing a 2.5% reduction: *The authors describe reduction in rotavirus prevalence, which are the comparative data from the country?*

“Though the two studies were carried out in the same location, of note is the fact that there was an increase in rotavirus awareness in the area (78% compared to 55.7% earlier reported) which might be due to the awareness created from the earlier study. Soleh, (2011) has suggested that increased awareness contribute to a child’s wellbeing in terms of preventing diseases like diarrhoea. Hence, the slight deviation in prevalence could be as a result of an increase in the level of awareness and enlightenment among parents and caregivers about childhood viral diarrhoea and its management”: *Please review, what are the data that the authors bring to support this social behavior? As this was not evaluated in the study?*

“Till date, rotavirus vaccine is yet to be introduced into the free Nigeria national immunization program but is still being paid for by parents who can afford it. Low prevalence of RVA has been reported by countries where RVA vaccine is fully introduced into their national immunization scheme. As suggested by Japhet et al. (2018), Nigeria needs to consider including rotavirus vaccine into the free immunization given to children.”: *Please consider to review the verb mode, as vaccine was introduced in August 2022.*
