# Supplementary material for: Rotavirus and bacterial diarrhoea among children in Ile-Ife, Nigeria: Burden, risk factors and seasonality
Source: PLoS One. 2023 Sep 12;18(9):e0291123. doi: 10.1371/journal.pone.0291123 (PMC10497142; doi:10.1371/journal.pone.0291123)
Supplement: Attachment — Submitted filename: Responses to comments by the Reviewer.docx [file pone.0291123.s005.docx]

Dear Academic Editor, PLOS ONE

Thank you for your invitation to review the second version of the manuscript “Rotavirus and bacterial diarrhoea among children in Ile-Ife, Nigeria: burden, clinical/environmental factors and seasonality”. The manuscript describes a basic hospital investigational of diarrhoea. The authors improved several issues that were pointed, however, there is still need to correct grammar issues throughout the entire manuscript as well as standardize the English language in British or United states. The authors should consider to review the following issues which were still not addressed.

1. **Abstract**

It was suggested to shorten and synthetize the abstract, however the authors reduced the amount of text making the background section lose its logic, the prior version of the background was better than the actual.

Response: The background section of the abstract has been reconstructed to retain its logic as in the prior version of the manuscript

2. **Introduction**
   2.1. The authors updated the data regarding diarrheal burden, using a new reference: WHO, 2019, however, the number of deaths was not updated with the actual data from the reference which states: “Diarrhoeal disease is the second leading cause of death in children under five years old, and was responsible for the deaths of 370,000 children in 2019. “, please correct. We suggest to maintain the phrase “The UNICEF”, instead of using “While the UNICEF”, as this contradicts the first affirmation.

Response: We apologize for this oversight. The mortality rate reported in the recent publication cited has been updated and each affirmation separated.

2.2. The statement “Considering the immaturity of children’s immune system, these poor living conditions increase the vulnerability of children younger than 5 years to diarrhoea when exposed to pathogens that cause diarrhoea” was not reviewed accordingly.

Response: The statement has been reviewed accordingly.

2.3. “Three major groups of microorganisms have been implicated as causal agents of diarrhoea. They include viruses (rotaviruses, caliciviruses, astroviruses and adenoviruses), bacteria (enterotoxigenic *Escherichia coli* (ETEC), enteropathogenic *Escherichia coli* (EPEC), *Shigella species, Salmonella species, Campylobacter jejuni*), and protozoa or parasites (*Entamoebahistolytica, Giardia lambia*) (7–9).” Replace ETEC and EPEC by DEC; and choose protozoa or parasites.

Response: We appreciate the reviewer for this comment. We have replaced ETEC and EPEC with DEC to cover both accordingly. We chose to retain parasite; hence protozoan has been expunged from the sentence.

2.4. “In Nigeria, there is dearth of single-study data on importance of rotavirus and bacteria as diarrhoea etiology in children, the associated burden, environmental/risk factors and the seasonality.” Is there enough data reporting the role of parasites in diarrhea in your setting?

Response: The focus of our study is on viruses and bacteria. The importance of the three major diarrhoea pathogens could be the focus of further research.

2.5. “It will inform appropriate policies for diarrhoea prevention and reduce indiscriminate antibiotics use, hence improving diarrhoea management in children.” The data from this study is not enough to support this affirmation.

Response: The data from our study serve as baseline data. This makes it pivotal and highly instrumental in making policies as regards diarrhoea prevention in the country, where such data does not even exist.

2.6. “…..Antibiotic susceptibility testing of DEC isolates was done on Mueller Hinton agar, using the disc diffusion method…….” This technique is standard and not need to be described in detail.

Response: We are grateful for the insight, redundant details on standard techniques have been removed.

2.7. “The DNA of *the E. coli* isolates were extracted using the boiling method. Briefly, three distinct colonies of each bacteria isolate were suspended in 100μl distilled water in a clean Eppendorf tube until emulsification was achieved; the resulting mixture was boiled at 100℃ for 15 minutes and finally centrifuged at 10,000 revolutions per minutes for five minutes in a microcentrifuge.” The authors performed a pull of three colonies?

Response: No, distinct colonies of each isolate were suspended; we have thus removed the number attached to the statement.

2.8. For AST interpretation the authors used CLSI 2018, consider to replace by the CLSI 2019 as the study was conducted between 2019 and 2020.

Response: We have interpreted our results using the CLSI 2019 guide.

2.9. “*Molecular Identification of Isolates by Amplification of 16SrRNA Gene*”. For clarification include *E. coli* in this subtitle.

Response: This has been done. Thank you.

2.10. “Rotavirus antigen detection was carried out by ELISA, using Human Rotavirus Antigen (RV-Ag) ELISA KIT (EKHU-1933, Melsin Medical Co., Ltd-Changchun, China), following the manufacturer's guide ([www.melsin.com/index.php?p=products_show&id=4308&lanmu=)](http://www.melsin.com/index.php?p=products_show&id=4308&lanmu=)).” Remove this link.

Response: We appreciate the reviewer for this comment. However, I am afraid we may not be able to remove the link, because this was specifically requested for by the other reviewer of our manuscript. The link was not included in the first manuscript that we submitted for review but was included in the corrected version in response to the request of one of the reviewers to have it included. Removing it now will mean we are editing the approved version by that reviewer

2.11. “Pathogen positive and negative samples were compared in terms of gender, age-group, seasonal distribution, and clinical/risk factors using Chi-square with two-tailed significance.” For those case in which the expected observation was less than five (small sample size) the authors did not consider the value of Fischer exact test?

Response: Thank you for the observation. We could not consider the use of Fischer exact test for these values because the data in question were not quantitative but qualitative (e.g yes or no; high or low, admitted not admitted etc)

3. **Results**
   3.1. The issues below raised before were not corrected accordingly:

Additionally, all the subtitles should be reviewed, authors describe them as if they were giving a conclusion or the main finding, ignoring the whole data in the section.

Rotavirus infection occurred only in the dry season: “Samples were not collected in December 2019 and March 2020 due to the outbreak of the Lassa fever virus in Nigeria and the outbreak of the global coronavirus pandemic which brought about restrictions in gaining access to the hospital and made parents to avoid coming to the hospital”: This information should have been described in the method section and authors should adjust the study period.

Response: We appreciate our reviewer for this comment and apologize for not addressing the issues raised totally. We have included the information about samples not been collected in parts of December and March in the method section as suggested. We do not think it necessary to adjust the study period since the period of no sample collection did not cover the whole of December and March. We actually had access to the facilities at the beginning of these months. We would have gotten samples if there were cases during those few periods. We mentioned this because we deemed it fit for transparency and good research ethics.

With regards to suggestion about the subtitles in our result section, we thank the reviewer for this. However, we adopted a style of writing found in various published articles, where authors highlight major findings in the result section without downplaying other information under such subheadings. A few of different articles in different journals with same style of writing are listed below:

1. Nitiema LW, Nordgren J, Ouermi D, Dianou D, Traore AS, Svensson L, Simpore J (2011). Burden of rotavirus and other enteropathogens among children with diarrhea in Burkina Faso. Int J Infect Dis. 15(9):e646-52. doi: 10.1016/j.ijid.2011.05.009.
2. Margaret Oluwatoyin Japhet, Adeyemi Adesina O, Famurewa O, Svensson L, Nordgren J (2012). Molecular epidemiology of rotavirus and norovirus in Ile-Ife, Nigeria: high prevalence of G12P[8] rotavirus strains and detection of a rare norovirus genotype. J Med Virol. 84(9):1489-96. doi: 10.1002/jmv.23343. PMID: 22825829.
3. Bucardo F, Reyes Y, Becker-Dreps S, Bowman N, Gruber JF, Vinjé J, Espinoza F, Paniagua M, Balmaseda A, Svensson L, Nordgren J (2017). Pediatric norovirus GII.4 infections in Nicaragua, 1999-2015. Infect Genet Evol. 55:305-312. doi: 10.1016/j.meegid.2017.10.001.
4. Hopkins FR, Govender M, Svanberg C, Nordgren J, Waller H, Nilsdotter-Augustinsson Å, Henningsson AJ, Hagbom M, Sjöwall J, Nyström S, Larsson M (2023). Major alterations to monocyte and dendritic cell subsets lasting more than 6 months after hospitalization for COVID-19. Front Immunol. 2023 Jan 4;13:1082912. doi: 10.3389/fimmu.2022.1082912.
   3.2. Percentages and *p-value* are missing in Table 3. Pathogens names should be italicized. Percentages missing in Table 4.

Response: We greatly appreciate the reviewer for the input on this. The pathogen names have been italicized. Percentage has been added to Table 4. We need to make it clear that the purpose of table 3 and table 4 are not to crosstab any variable, hence no p-value was generated.

3.3. “Twenty-four *E*. *coli* pathogroups belonging to four *E*. *coli* pathotypes were detected in the study. Of the 24 *E*. *coli* pathogroups detected, majority of the isolates belong to the Shiga Toxin *E. coli* (STEC) while some were found to have cross bands of pathogenic genes. Nineteen (79.2%) of the isolates were STEC, one isolate (4.2%) was found to be an EAEC while 4 (16,7%) isolates had a cross band of genes (stx1/stx2 and CVD342) belonging to STEC and EAEC as shown in Table 4.” I don’t understand when the authors say pathogroups. In addition, this section has redundant information.

Response: We referred to Diarrhoegenic *E. coli* as pathogroups. To avoid ambiquity and to give proper meaning, we have replaced the pathogroups with DEC accordingly.

3.4. “The isolates showed 100% resistance to the Cephalosporin (cefoxitin, cefuroxime sodium), while 95.8% resistance was observed with the Sulfonamide (sulphamethoxazole/Trimethoprim).” Why while if 100% is close to 95.8%.

Response: As pointed out by the reviewer, we have removed “while” since the gap between the two classes were close, hence no need to have a comparison. Thank you.

3.5. Figure 2 only shows absolute number, there is no information of antimicrobial susceptibility rates. Same for Figure 3.

Response: Antimicrobial susceptibility rates has been added to the figure.

3.6. “…..were detected among the 43 females. but acute gastroenteritis (AGE) infection was not sex-related (P> 0.05)…..” Please check this sentence

Response: We have corrected the punctuation error in the sentence highlighted above.

3.7. “….High knowledge about RVA was observed among the parent/guardian. Specifically, 75.8% (69/91) of the parents have knowledge about the virus…..” What the authors mean with “high knowledge”?

Response: We referred to parents who were aware of rotavirus as the cause of diarrhoea in the children as those with “high Knowledge”. We have corrected the statement to reflect this. Thank you.

3.8. “childAnthropometric measurement (mid-upper arm circumference measurement), indicative of low nutritional status…” Please check this sentence

Response: We have checked the sentence and discover a space is missing in between child and anthropometric. This has been adjusted appropriately. Thank you.
