# Supplementary material for: Rotavirus and bacterial diarrhoea among children in Ile-Ife, Nigeria: Burden, risk factors and seasonality
Source: PLoS One. 2023 Sep 12;18(9):e0291123. doi: 10.1371/journal.pone.0291123 (PMC10497142; doi:10.1371/journal.pone.0291123)
Supplement: Attachment — Submitted filename: PONE-D-22-22211_Reviewer letter.docx [file pone.0291123.s006.docx]

Date: 02 April 2023

To: PLOS ONE

Subject: Review of the manuscript – PONE-D-22-22211R1

Dear Academic Editor, PLOS ONE

Thank you for your invitation to review the third version of the manuscript “Rotavirus and bacterial diarrhoea among children in Ile-Ife, Nigeria: burden, clinical/environmental factors and seasonality”. The manuscript describes a basic hospital investigational of diarrhoea. The authors have improved several issues that were pointed, however, there is still need to correct the abstract:

1. **Abstract**

**Background**: **Suggestion**: “Diarrhea is a leading cause of death among under-five children globally, with sub-Saharan Africa alone accounting for 1/3 episodes yearly. Viruses, bacteria and parasites may cause diarrhea. Rotavirus is the most common viral etiology of diarrhoea in children less than five years globally. In Nigeria, there is scarce data on the prevalence/importance, burden, clinical/risk factors and seasonality of rotavirus and bacteria and this study aims to determine the role of rotavirus and bacteria on diarrheal cases in children less than five years in Ile-Ife, Nigeria.

**Methods**: Consider removing ..from 104 under five, because seems to be a result

**Conclusion**: **Suggestion**: In this study, rotavirus was more prevalent than bacteria and occurred only in the dry season. Among bacterial etiologies, *E. coli*. was the most common detected. Differences in seasonal peaks of rotavirus and *E.coli* could be employed in diarrhoea management in Nigeria and other tropical countries for optimal usage of limited resources usage in preventing diarrhoea transmission and reducing indiscriminate antibiotics usage. **Notice that the word indiscriminate is wrongly written in your conclusion. *E. coli* must be replaced by DEC.**
