# Supplementary material for: Rotavirus and bacterial diarrhoea among children in Ile-Ife, Nigeria: Burden, risk factors and seasonality
Source: PLoS One. 2023 Sep 12;18(9):e0291123. doi: 10.1371/journal.pone.0291123 (PMC10497142; doi:10.1371/journal.pone.0291123)
Supplement: Attachment — Submitted filename: Response to comments by Reviewer_April 2023.docx [file pone.0291123.s007.docx]

Date: 11 April 2023

To: PLOS ONE

Subject: Response to Reviewer’s comment on the manuscript – PONE-D-22-22211R1

**Academic Editor’s comment**

If applicable, we recommend that you deposit your laboratory protocols in [protocols.io](http://protocols.io/) to enhance the reproducibility of your results.

Response: The recommendation to deposit our protocol is not applicable to this study.

Please review your reference list to ensure that it is complete and correct. If you have cited papers that have been retracted, please include the rationale for doing so in the manuscript text, or remove these references and replace them with relevant current references. Any changes to the reference list should be mentioned in the rebuttal letter that accompanies your revised manuscript. If you need to cite a retracted article, indicate the article’s retracted status in the References list and also include a citation and full reference for the retraction notice.

Response: We have carefully reviewed all our references, all the references cited in our manuscript are still available online, none is retracted.

Journal Requirements: Please upload your figure files to the Preflight Analysis and Conversion Engine (PACE) digital diagnostic tool, <https://pacev2.apexcovantage.com/>. PACE helps ensure that figures meet PLOS requirements.

Response: All our figures have been uploaded to the preflight analysis and conversion engine (PACE) as requested.

**Reviewer’s comment**

Again, we appreciate the invaluable suggestions of our indefatigable reviewer towards making our manuscript to be at its best. The suggestions on the abstract have been accepted and addressed below with slight modification to avoid repeated use of the word “usage” in a single paragraph.

1. **Abstract**

**Background**: **Suggestion**: “Diarrhea is a leading cause of death among under-five children globally, with sub-Saharan Africa alone accounting for 1/3 episodes yearly. Viruses, bacteria and parasites may cause diarrhea. Rotavirus is the most common viral etiology of diarrhoea in children less than five years globally. In Nigeria, there is scarce data on the prevalence/importance, burden, clinical/risk factors and seasonality of rotavirus and bacteria and this study aims to determine the role of rotavirus and bacteria on diarrheal cases in children less than five years in Ile-Ife, Nigeria.

Response: This has been accepted and effected.

**Methods**: Consider removing ..from 104 under five, because seems to be a result

Response: This has been removed. Thank you.

**Conclusion**: **Suggestion**: In this study, rotavirus was more prevalent than bacteria and occurred only in the dry season. Among bacterial etiologies, *E. coli*. was the most common detected. Differences in seasonal peaks of rotavirus and *E.coli* could be employed in diarrhoea management in Nigeria and other tropical countries for optimal usage of limited resources usage in preventing diarrhoea transmission and reducing indiscriminate antibiotics usage.

Response: To avoid repeated use of the word “usage” which appeared thrice in the reviewer’s suggestion in a single paragraph, we reconstructed the conclusion thus:

In this study, rotavirus was more prevalent than bacteria and occurred only in the dry season. Among bacteria aetiologies, DEC was the most common detected. Differences in seasonal peaks of rotavirus and DEC could be employed in diarrhoea management in Nigeria and other tropical countries to ensure optimal usage of limited resources in preventing diarrhoea transmission and reducing indiscrimate use of antibiotics.

**Notice that the word indiscriminate is wrongly written in your conclusion. *E. coli* must be replaced by DEC.**

Thank you for your observation, we have now correctly spelt ‘indiscriminate’ and *E.coli* has been replaced with DEC.
