# Supplementary material for: Rotavirus and bacterial diarrhoea among children in Ile-Ife, Nigeria: Burden, risk factors and seasonality
Source: PLoS One. 2023 Sep 12;18(9):e0291123. doi: 10.1371/journal.pone.0291123 (PMC10497142; doi:10.1371/journal.pone.0291123)
Supplement: Attachment — Submitted filename: Reviewer comment.docx [file pone.0291123.s004.docx]

Dear Academic Editor, PLOS ONE

Thank you for your invitation to review the second version of the manuscript “Rotavirus and bacterial diarrhoea among children in Ile-Ife, Nigeria: burden, clinical/environmental factors and seasonality”. The manuscript describes a basic hospital investigational of diarrhoea. The authors improved several issues that were pointed, however, there is still need to correct grammar issues throughout the entire manuscript as well as standardize the English language in British or United states. The authors should consider to review the following issues which were still not addressed.

1. **Abstract**

It was suggested to shorten and synthetize the abstract, however the authors reduced the amount of text making the background section lose its logic, the prior version of the background was better than the actual.

2. **Introduction**
   2.1. The authors updated the data regarding diarrheal burden, using a new reference: WHO, 2019, however, the number of deaths was not updated with the actual data from the reference which states: “Diarrhoeal disease is the second leading cause of death in children under five years old, and was responsible for the deaths of 370,000  children in 2019. “, please correct. We suggest to maintain the phrase “The UNICEF”, instead of using “While the UNICEF”, as this contradicts the first affirmation.
   2.2. The statement “Considering the immaturity of children’s immune system, these poor living conditions increase the vulnerability of children younger than 5 years to diarrhoea when exposed to pathogens that cause diarrhoea” was not reviewed accordingly.
   2.3. “Three major groups of microorganisms have been implicated as causal agents of diarrhoea. They include viruses (rotaviruses, caliciviruses, astroviruses and adenoviruses), bacteria (enterotoxigenic *Escherichia coli* (ETEC), enteropathogenic *Escherichia coli* (EPEC), *Shigella species, Salmonella species, Campylobacter jejuni*), and protozoa or parasites (*Entamoebahistolytica, Giardia lambia*) (7–9).” Replace ETEC and EPEC by DEC; and choose protozoa or parasites.
   2.4. “In Nigeria, there is dearth of single-study data on importance of rotavirus and bacteria as diarrhoea etiology in children, the associated burden, environmental/risk factors and the seasonality.” Is there enough data reporting the role of parasites in diarrhea in your setting?
   2.5. “It will inform appropriate policies for diarrhoea prevention and reduce indiscriminate antibiotics use, hence improving diarrhoea management in children.” The data from this study is not enough to support this affirmation.
   2.6. “…..Antibiotic susceptibility testing of DEC isolates was done on Mueller Hinton agar, using the disc diffusion method…….” This technique is standard and not need to be described in detail.
   2.7. “The DNA of *the E. coli* isolates were extracted using the boiling method. Briefly, three distinct colonies of each bacteria isolate were suspended in 100μl distilled water in a clean Eppendorf tube until emulsification was achieved; the resulting mixture was boiled at 100℃ for 15 minutes and finally centrifuged at 10,000 revolutions per minutes for five minutes in a microcentrifuge.” The authors performed a pull of three colonies?
   2.8. For AST interpretation the authors used CLSI 2018, consider to replace by the CLSI 2019 as the study was conducted between 2019 and 2020.
   2.9. “*Molecular Identification of Isolates by Amplification of 16SrRNA Gene*”. For clarification include *E. coli* in this subtitle.
   2.10. “Rotavirus antigen detection was carried out by ELISA, using Human Rotavirus Antigen (RV-Ag) ELISA KIT (EKHU-1933, Melsin Medical Co., Ltd-Changchun, China), following the manufacturer's guide ([www.melsin.com/index.php?p=products_show&id=4308&lanmu=)](http://www.melsin.com/index.php?p=products_show&id=4308&lanmu=)).” Remove this link.
   2.11. “Pathogen positive and negative samples were compared in terms of gender, age-group, seasonal distribution, and clinical/risk factors using Chi-square with two-tailed significance.” For those case in which the expected observation was less than five (small sample size) the authors did not consider the value of Fischer exact test?
3. **Results**
   3.1. The issues bellow raised before were not corrected accordingly:

Additionally, all the subtitles should be reviewed, authors describe them as if they were giving a conclusion or the main finding, ignoring the whole data in the section.

Rotavirus infection occurred only in the dry season: “Samples were not collected in December 2019 and March 2020 due to the outbreak of the Lassa fever virus in Nigeria and the outbreak of the global coronavirus pandemic which brought about restrictions in gaining access to the hospital and made parents to avoid coming to the hospital”: This information should have been described in the method section and authors should adjust the study period.

3.2. Percentages and *p-value* are missing in Table 3. Pathogens names should be italicized. Percentages missing in Table 4.
3.3. “Twenty-four *E*. *coli* pathogroups belonging to four *E*. *coli* pathotypes were detected in the study. Of the 24 *E*. *coli* pathogroups detected, majority of the isolates belong to the Shiga Toxin *E. coli* (STEC) while some were found to have cross bands of pathogenic genes. Nineteen (79.2%) of the isolates were STEC, one isolate (4.2%) was found to be an EAEC while 4 (16,7%) isolates had a cross band of genes (stx1/stx2 and CVD342) belonging to STEC and EAEC as shown in Table 4.” I don’t understand when the authors say pathogroups. In addition, this section has redundant information.
3.4. “The isolates showed 100% resistance to the Cephalosporin (cefoxitin, cefuroxime sodium), while 95.8% resistance was observed with the Sulfonamide (sulphamethoxazole/Trimethoprim).” Why while if 100% is close to 95.8%.
3.5. Figure 2 only shows absolute number, there is no information of antimicrobial susceptibility rates. Same for Figure 3.
3.6. “…..were detected among the 43 females. but acute gastroenteritis (AGE) infection was not sex-related (P> 0.05)…..” Please check this sentence
3.7. “….High knowledge about RVA was observed among the parent/guardian. Specifically, 75.8% (69/91) of the parents have knowledge about the virus…..” What the authors mean with “high knowledge”?
3.8. “childAnthropometric measurement (mid-upper arm circumference measurement), indicative of low nutritional status…” Please check this sentence
